# Impact of Biochar Application at Water Shortage on Biochemical and Physiological Processes in *Medicago ciliaris*

**DOI:** 10.3390/plants11182411

**Published:** 2022-09-15

**Authors:** Jihed Gharred, Walid Derbali, Imed Derbali, Mounawer Badri, Chedly Abdelly, Inès Slama, Hans-Werner Koyro

**Affiliations:** 1 Institute of Plant Ecology, Justus Liebig University Giessen, 35392 Giessen, Germany; 2 Laboratory of Extremophile Plants, Center of Biotechnology of Borj Cedria, Hammam-Lif 2084, Tunisia; 3 Faculty of Mathematical, Physical and Natural Sciences of Tunis, University of Tunis EL-Manar, Tunis 1068, Tunisia

**Keywords:** drought, *M. ciliaris*, biochar, growth, photosynthesis, antioxidant capacity

## Abstract

The application of biochar is mostly used to improve soil fertility, water retention capacity and nutrient uptake. The present study was conducted in order to study the impact of biochar at water deficiency conditions on the physiological and biochemical processes of *Medicago ciliaris* seedlings. Seedlings were cultivated under greenhouse conditions in pots filled with a mixture of soil and sand mixed in the presence or absence of 2% biochar. Plants of uniform size were subjected after a pretreatment phase (72 days) either to low (36% water holding capacity, water potential low) or high soil water potential (60% water holding capacity, water potential high). Pots were weighed every day to control and maintain a stable water holding capacity. In *Medicago ciliaris*, drought led to a significant reduction in plant growth and an increase in the root/shoot ratio. The growth response was accompanied by a decreased stomatal conductance and a reduction of the net CO_2_ assimilation rate and water use efficiency. The associated higher risk of ROS production was indicated by a high level of lipid peroxidation, high antioxidant activities and high proline accumulation. Soil amendment with biochar enhanced the growth significantly and supported the photosynthetic apparatus of *Medicago ciliaris* species by boosting chlorophyll content and A_net_ both under well and insufficient watered plants and water use efficiency in case of water shortage. This increase of water use efficiency was correlated with the biochar-mediated decrease of the MDA and proline contents in the leaves buffering the impact of drought on photosynthetic apparatus by increasing the activity of enzymatic antioxidants SOD, APX, GPOX and GR and non-enzymatic antioxidants, such as AsA and DHAsA, giving the overall picture of a moderate stress response. These results confirmed the hypothesis that biochar application significantly reduces both the degree of stress and the negative impact of oxidative stress on *Medicago ciliaris* plants. These results implied that this species could be suitable as a cash pasture plant in the development of agriculture on dry wasteland in a future world of water shortages.

## 1. Introduction

In the next few decades, experts estimate that there will be a rise in global temperature of around 1.5 to 2 °C [1]. The cold seasons will become shorter and the warm seasons will become longer and heatwaves will occur more often, according to the report released by the Intergovernmental Panel on Climate Change [1]. Warmer temperatures enhance evaporation, which reduces surface water and dries out soils and vegetation. Nevertheless, the intensity of drought usually depends on many factors, such as the occurrence and distribution of rainfall, shifting seasons, evaporative demands and the moisture-storing capacity of the soil, especially in semiarid climates [2,3]. Facing fluctuating and unstable environmental conditions, plants need to adjust to these changes by relying on their resilience (exposure, sensitivity, adaptive capacity) and vulnerability [4]. This includes a response to water and nutrient shortage, temperature variations, UV radiation, fungal pathogens and insects, as well as other biotic and abiotic factors [5,6].

Drought impacts are not confined to arid and semi-arid regions but are increasingly spreading to more temperate and humid areas [7]. Although droughts affect a range of economically important sectors, their impacts are usually more evident within agriculture, including crop failure and reduced yields, abandoned farmland, increased soil degradation and reduced mortality [7,8]. This situation creates an urgent need for attaining agricultural sustainability regarding building resilience and adaptive capacity. Agricultural strategies are required, such as the efficient use of irrigation (= increasing water productivity (WP)), increasing livestock production relative to crops and the selection of alternative crop varieties. This may also open the possibility of enhancing productivity and food security in marginal environments (e.g., deserted or salinized regions) through the contribution of alternative crops [9,10,11].

Preferably, endemic plants should be chosen to ensure sustainability and environmental compatibility on dry wasteland. Some representatives of the family Fabaceae and genus *Medicago* are suitable candidates. They are high-quality, protein-rich food sources with a low demand for nutrients, a distinct resistance to saline conditions and the ability to grow on low-quality sandy soils [11,12]. *Medicago* is already in use as an alternative crop because of its ecological adaptability, morphological diversity, resistance to pests, high forage quality, high biomass production and ability to reduce soil erosion and to improve soil fertility and pasture in arid Mediterranean regions [13,14,15,16]. The latter species is caused by symbiotic nitrogen fixation, leading to a rise of the nitrogen content in plant and soil [17,18]. However, there is a high variation of drought resistance in this genus [19]. Badri [20] studied the variation in tolerance to water deficit in 47 lines of *Medicago truncatula Gaertn.*, *M. polymorpha* L. and *M. ciliaris* (L.) All. The latter was the latest-flowering and produced the highest biomass at low soil water availability. Therefore, we selected *Medicago ciliaris* as the test species.

The suitability of *Medicago ciliaris* depends on the efficiency of its strategies during the adaptation to water shortage. Volaire [4] proposed the existence of three primary plant eco-physiological strategies: avoidance, tolerance and escape.

Desiccation tolerance is relatively seldom in dicot plants and *Medicago ciliaris* does not have this ability. In dicots, only resurrection plants are a small polyphyletic group of plant species capable of desiccation tolerance in their vegetative tissues while being able to recover full metabolic competence within hours to days following rehydration [21]. Therefore, *Medicago ciliaris* seems to use the escape or avoidance strategy or a mixture of both [22]. Instead of desiccation tolerance, and as a first feedback reaction during transient periods of drought stress, both avoider and escaper plants respond as follows: the reduction of transpiration, the limitation of vegetative growth, the enhancement of root growth and the avoidance of dehydration [23].

Both remaining strategies also have in common a large energy demand; the necessity to optimize water uptake and to minimize water loss by a high water use efficiency of photosynthesis and during osmotic adjustment; the osmo-protection of macromolecules; the limitation of temperature rise in the leaf; the control of the respiration rate; and the protection against reactive oxygen species (ROS).

In fact, the decreased diffusion of CO_2_ and metabolic constraints affect photosynthesis as one of the key phenomena of water deficiency [24]. Photosynthesis is a decisive point of attack and, at the same time, the key process controlling plant growth and development and thus crop yield. Water shortage, for example, can reduce photosynthesis in plants through stomatal and non-stomatal limitations [25], but the coordinated regulation of photosynthesis in plants can increase biomass production and resistance to environmental stress [26].

Water shortage can harm photosynthesis directly through the restriction of CO_2_ uptake, which leads to damage in photosynthetic machinery and, as a final consequence, to the development of reactive oxygen species (ROS) [26,27]. As oxygen is produced by the water-splitting system located adjacent to PSII, ROS formation is a real risk. Therefore, the degree of ROS damage and the efficiency of the ROS detoxification system in the chloroplasts are of crucial importance for survival.

In fact, ROS has a high affinity to react with macromolecules, such as lipids, proteins and nucleic acids, and cause the malfunctioning of these macromolecules [28]. For this reason it is very helpful that the extent of ROS damage can be estimated by measuring the peroxidation rate of membrane lipids with the malondialdehyde method [29,30].

However, in order to cope with an increased ROS production, plant cells display a complex and high-energy consuming array of both enzymatic and non-enzymatic detoxification mechanisms [31]. The latter group includes the production of low-molecular weight compounds, such as AsA (ascorbate, vitamin C), glutathione (GSH), carotenoids or flavonoids [32,33]. AsA is one of the most abundant water-soluble reducing compounds present in plant tissues, serving also as an electron donor in numerous reactions [34], such as the effective quenching of H_2_O_2_ [35]. In addition, the roles of non-photochemical fluorescence quenching parameters (NPQ), cycling electron flow (CEF), Foyer–Halliwell–Asada cycle and the repair cycle for damaged PSII reaction centers in photoprotection are well established [36,37,38].

In order to carry out a successful recultivation at low soil water availability, it is necessary to improve soil quality and thus the ability of plants species to resist this harsh environment.

A means to improving soil quality is the amendment of biochar (Bc). Bc can significantly increase organic matter content, water holding capacity and the plant-available water in poor-quality sandy soil, the latter characteristic due to Bc’s porous nature [26,39]. The addition of Bc to the soil proved to be beneficial to plants in many ways, such as enhancing water retention capacity, nutrient uptake [40,41,42] water infiltration [43,44], soil aeration and respiration [42,45]. Moreover, Bc-stimulated root growth and thus water uptake from fine Bc pores. It also provided at low water supply better conditions for the synthesis of organic solutes, prevented desiccation with improved turgidity and reduced oxidative stress through high water use efficiency [46,47].

The soil improvements with Bc jointly contribute to the increase on the physiological and biochemical performances of plants and consequently promote plant biomass production. These beneficial effects of Bc were reported in several species, such as maize and rice [48].

However, it should be noted that the effects of biochar under drought conditions on water relations are contradictory. For instance, in soybean [49], authors reported that Bc application significantly enhanced crop growth rate and increased total biomass production approximately twice. Bc also improved WUE but did not improve relative water content (RWC), water retention or uptake capacity in leaves. The authors explained the enhanced biomass production by an improvement in plant nutrition rather than by increasing water uptake. Indeed, Bc application increased soil-available potassium (K) and enhanced its uptake, which lead to an increase in the stress tolerance of soybean.

Lyu reported that the plant defense mechanism is strengthen by Bc application through the increase in protective enzymatic activities and the electron transfer chain, thereby minimizing the deleterious effects of drought on the photosynthetic apparatus [50].

Currently, no data in the literature are available regarding the effectiveness of Bc on the response of *Medicago ciliaris* seedlings to drought stress. It is our hypothesis that Bc biochar application significantly reduces both the degree of stress and the negative impact of oxidative stress on *Medicago ciliaris* plants. It was our intension to study the impact of drought and Bc on the photosynthesis, water use efficiency, oxidative stress damage, ROS defense and growth performance of this promising species. Moreover, regarding the study of the physiological and biochemical mechanisms implied in the response of *Medicago ciliaris* to water shortage, our aim is to improve the response of this species to water deficit through the addition of Bc to the culture medium and to increase the productivity of alternative crops through the development of agriculture on dry wasteland to transform unproductive areas.

## 2. Materials and Methods

### 2.1. Plant Material and Growth Conditions

The *Medicago ciliaris* line used in this work was kindly provided by the Laboratory of Extremophile Plants in the Center of Biotechnology at The Technopole of Borj Cedria in Tunisia. This line originated from a local Tunisian population of Enfitha (TNC1.11). In *Medicago ciliaris*, germination is strongly limited by the presence of a hard seed coat; thus, to obtain a maximal rate of germinated seeds, scarification with liquid nitrogen was necessary.

Scarified seeds were germinated in Petri dishes in dark at 25 °C for 3 days then transferred into black pots with a 1.55 L capacity (pot 13 × 13 cm; dimensions at top: 13 × 13 cm; dimensions at the bottom: 9.5 × 9.5 cm, height: 12.5 cm) filled with a mixture of soil (70%) and sand (30%), either mixed or not mixed with 2% Bc. Coniferous wood and hardwood chips (1:4 ratio by weight) were mixed to produce Biochar through pyrolysis in a 36-h cycle at 750 °C using a Schottdorf-type reactor (Carbon Terra, Augsburg, Germany). The experiment was carried out at the University of Giessen, Germany, in a controlled environment greenhouse equipped with an automated greenhouse climate control system (including air conditioner) at a temperature of 24 °C/15 °C (day/night), a relative humidity of 55–60% and a photoperiod of 16/8 h. The emerged seedlings of uniform size were subjected to pretreatment phase (72 days). Two irrigation modes were retained in this study at 60 and 36% WHC. The selection of this two percent of water-holding capacity was based on a preliminary experiment carried out on *Medicago ciliaris* watered with 100, 75, 60 and 36% WHC, showing that for our line and soil type, 60% corresponded to the optimal conditions for growth and that 36% WHC led to a significant decrease of growth parameters. Thus, plants irrigated with 60% WHC corresponded to control plants; however, plants irrigated with 36% WHC correspond to stressed ones. Pots were weighed every day to control and maintain a stable WHC

For all treatments, water was enriched with diluted nutrient solution [51]. Independently of the procedure for watering (60 or 36% WHC), plants received the same quantity of nutrients.

WHC measured according to the technique of Bouyoucos [52] was estimated at around 13.33%.

After 42 days of treatment, a final harvest was carried out and plants were separated into shoots and roots.

### 2.2. Growth Parameter

Upon harvest, we measured root, shoot and nodule fresh weight (FW) separately. Dry weight (DW) was determined after drying the samples at 60 °C in the oven until a constant weight was reached.

Sensitivity index (SI) was also determined by measuring the difference between the DW of plants subjected to water deficit stress and control plants and the DW of the controls according to the following equation expressed in percent [53]:

**SI_stress_ = [100 × (DW_stressed_ − DW_control_)/DW_control_]** (1)

#### 2.2.1. Chlorophyll Fluorescence

Chlorophyll fluorescence was measured in the third fully expanded leaf from starting in the morning from 08:30 am to 15:00 am, using a portable chlorophyll meter (JUNIOR PAM, WALZ GmbH, Effeltrich, Germany). Before measuring, leaves needed to be adapted to the darkness for 40 min to evaluate maximum quantum efficiency of PSII photochemistry [54]. Fluorescence parameters characterizing either the dark-adapted state or light-adapted state were measured at four plants from each of the four applied treatments.

The following chlorophyll fluorescence parameters were calculated using WINCONTROL software (2.133/03.00) with standard settings for rapid light curves (Heinz Walz Gmbh, Effeltrich, Germany; [55,56]), which are the potential maximal efficiency of PSII (Fv/Fm), electron transport rate (ETR), photochemical quantum yield of photosystem II (Y(II)); the quantum yield of regulated non-photochemical energy loss in PS II (Y(NPQ)), quantum yield of non-regulated non-photochemical energy loss in PS II, equivalent to Y(NO); and photosynthetic photon flux density (PPFD) (μE m^−2^ s^−1^).

#### 2.2.2. CO_2_/H_2_O Gas Exchange

CO_2_/H_2_O gas exchange was determined using a Li-Cor LI-6400XT portable photosynthesis system (Li-Cor Biosciences; Lincoln, NE, USA) with a 6400-02(B) LED light source attached to the leaf chamber.

Temperature in the leaf was set at 22.0 °C. Carbon dioxide levels in the leaf chamber were controlled by using CO_2_ cartridge and a fixed flow rate of 300 μmol s^−1^. CO_2_ concentration within the leaf chamber (C_a_) was fixed at 400 μmol mol^−1^. Intercellular CO_2_ concentration (C_i_) [μmol CO_2_ m^–2^ s^–1^], net CO_2_ assimilation rate (A_net_) [μmol CO_2_ m^–2^ s^–1^], dark respiration (R_D_) [μmol CO_2_ m^–2^ s^–1^], transpiration rate (E) [mol H_2_O m^–2^ s^–1^] and stomatal conductance (S_C_) [mol H_2_O m^–2^ s^–1^] were determined on the third fully expanded leaf from 08:30 to 15:00 am. Water use efficiency (WUE) was calculated as a A_net_/E ratio. Photorespiration (R_L_) [μmol(CO_2_) m^–2^ s^–1^] was estimated as 1/12 (ETR − 4 (A_net_ + RD)) [57]. Gross CO_2_ assimilation (A_gross_) [μmol (CO_2_) m^–2^ s^–1^] was calculated as the sum of A_net_, R_D_ and R_L_. The slope in the linear range of the light response curve represents the photosynthetic efficiency (V_c_) and was calculated as described in [58]. All measurements were carried out in the greenhouse at light saturation conditions with 750 or 1500 μE m^−2^ s^−1^ photosynthetic photon flux density (PPFD) (high water potential: 1500 µmol m^−2^ s^−1^ PPFD and low water potential: 750 µmol m^−2^ s^−1^ PPFD) with 25 ± 15 °C air temperature and 60 ± 10% relative air humidity.

#### 2.2.3. Chlorophyll Content

Leaf SPAD readings (SPAD 502; Minolta Co., Osaka, Japan) provide a nondestructive surrogate method for determining leaf chlorophyll (Chl) concentration [59]. Leaf chlorophyll (Chl) concentrations were measured in the third fully expanded leaf in the morning. The mean of three SPAD readings for each leaf was recorded.

#### 2.2.4. Proline Content

Free proline was qualified spectrophotometrically according to Bates [60].

An amount of 0.2 g of plant fresh material was homogenized in 4 mL of sulphosalicylic acid (3% *w*/*v*), then mixed with 2 mL of acid ninhydrin solution and 2 mL of glacial acetic acid. The mixture was heated at +100 °C for 1 h in a water bath. The reaction was stopped by transferring the mixture to an ice bath. Proline was extracted by adding 4 mL of toluene to each tube, and the absorbance of toluene fraction (aspired from the liquid phase) was measured at λ 520 nm using a UV/VIS spectrophotometer CAMSPEC M550 double beam (Spectronic CamSpec, Leeds, UK). Proline concentration was determined using calibration curve as μmol proline g^−1^ FW.

#### 2.2.5. Lipid Peroxidation

The extent of lipid peroxidation was estimated by determining the concentration of malondialdehyde (MDA) according to Rao and Sresty [61]. Leaf material (50 mg FW) was homogenized with a prechilled mortar and pestle in 2 mL of ice-cold trichloroacetic acid TCA (0.1%, *w*/*v*) and centrifuged at 15,000× *g* for 15 min and at 4 °C. Assay mixture containing 2 mL aliquot of supernatant and 2 mL of 0.67% (*w*/*v*) thiobarbituric acid (TBA), was heated at 95 °C for 20 min and then rapidly cooled in an ice bath. The samples were centrifuged (10,000× *g* for 10 min at 4 °C) and the supernatant absorbance was measured at λ 532 and λ 600 nm using UV/VIS spectrophotometer CAMSPEC M550 double beam (Spectronic CamSpec, Leeds, UK). The concentration of MDA was calculated from the extinction coefficient 155 mM^−1^ cm^−1^.

#### 2.2.6. Hydrogen Peroxide Content

The hydrogen peroxide (H_2_O_2_) concentration was measured according to the method previously described by Loreto and Velikova [62]. Frozen leaf samples (500 mg) were homogenized in 5 mL of 1% (*w*/*v*) ice-cold trichloroacetic acid (TCA) and centrifuged at 14,000× *g*, for 20 min at 4 °C. Subsequently, 0.5 mL of supernatant was mixed with 0.5 mL of potassium phosphate buffer (10 mM, pH 7.0) and 1.5 mL of potassium iodide (1 M) in a ratio 2:1 (*v*/*v*). The absorbance was measured at λ 390 nm using a UV/VIS spectrophotometer CAMSPEC M550 double beam (Spectronic CamSpec, Leeds, UK). The hydrogen peroxide content was calculated using a standard curve using different concentrations of H_2_O_2_.

### 2.3. Protein Quantification and Antioxidant Enzyme Assay

Fresh leaves (100 mg) were homogenized with ice-cold sodium phosphate buffer (50 mM, pH 7.2) containing 1 mM ascorbic acid, 1mM dithiothreitol (DTT), 0.1% of triton, 10 mM ethylene diamine tetra acetic acid (EDTA, disodium salt) and 10% (*w*/*v*) Polyvinylpolypyrrolidone (PVPP). The homogenate was centrifuged at 12,000× *g* for 20 min at 4 °C. The supernatant was collected and stored in small Eppendorf at −80 °C.

Protein content was determined after mixing the supernatant with an acid solution of Coomassie–Brillant–Blau G-250 and subsequent incubation in the dark for 10 min (see Bradford, 1976). The absorbance was measured at λ 595 nm using a UV/VIS spectrophotometer CAMSPEC M550 double beam (Spectronic CamSpec, Leeds, UK). Soluble protein concentration in the enzyme extracts were estimated using a standard curve of different concentrations of bovine serum albumin (BSA).

Superoxide dismutase activity was assayed by its ability to inhibit photochemical reduction of nitroblue tetrazolium chloride (NBT) at 560 nm. According to Beyer and Fridovich [63], we prepared a reagent containing 10 mM of L-methionine, 0.1 mM of nitroblue-tetrazolium chloride (NBT) and 0.75% of Triton X-100 in 50 mM potassium phosphate pH 7.8 in a dark bottle. Of this reagent, 1 μL was added to the reaction mixture (3 mL) containing 40 μL of enzyme extract followed by 10 μL of 0.12 mM riboflavin. The mixture was prepared twice, one of them was incubated under fluorescent lamps (40 W) for 7 min and the second was kept in the dark to be used as blank for the measurements. The absorbance of the mixture was measured at λ 560 nm. The enzyme activity was calculated as the percentage inhibition per minute.

Ascorbate peroxidase (APX, EC 1.11.1.11) activity was assayed according to Nakano and Asada [64]. The reaction mixture (3 mL) consisted of 50 mM of potassium phosphate buffer (pH 7.0), 0.2 μM of EDTA, 0.5 mM of ascorbate, 2 mM H_2_O_2_ and 50 μL of enzyme extract. The reaction was initiated by the addition of H_2_O_2_. Ascorbate peroxidase was assayed by monitoring the decrease in absorbance at 290 nm. The molar extinction coefficient was 2.8 mM^−1^ cm^−1^.

Guaiacol peroxidase (GPX) activity was measured by recording the increase of the absorbance at λ 470 nm due to a tetra-guaiacol formation (ε = 26.6 L mol^−1^ cm^−1^) according to Tatiana [65]. The reaction mixture (3 mL) contained 50 mM of potassium phosphate buffer (pH 7.0), 2 mM H_2_O_2,_ Guaiacol 2.7 mM and 50 μL of enzyme extract. The enzyme activity was calculated as the percentage of inhibition per min. The molar extinction coefficient was 26.6 L mol^−1^ cm^−1^.

According to Foyer and Halliwell [66], Glutathione reductase (GR, EC 1.6.4.2) activity was determined by the oxidation of β-NADPH at λ 340 nm (ε = 6.2L mol^−1^ cm^−1^). The reaction mixture (3 mL) contained 100 mM Tris-HCl (pH 7.8), 0.5 mM GSSG, 0.03 mM β-NADPH, 5 mM EDTA and 100 μL of enzyme extract. The molar extinction coefficient was 6.2 L mol^−1^ cm^−1^.

### 2.4. Extraction and Determination of Non-Enzymatic Antioxidant Ascorbate (AsA) and Dehydro-Ascorbate (DHAsA)

Frozen leaf samples (400 mg) were ground in liquid nitrogen and homogenized in 2 mL of ice cold 6% TCA. The mixture was centrifuged at 16,000× *g* for 20 min at 4 °C and supernatant was collected. Ascorbate (AsA) and dehydro-ascorbate (DHAsA) were determined with a dipyridyl assay based on the reduction of Fe^+3^ by reduced ascorbate, followed by complex formation between Fe^+2^ and bipyridil, which absorbs at λ 525 nm. Total ascorbate was determined after the reduction of DHAsA to AsA by reacting with dithiothreitol. A standard curve was prepared for the estimation of total ascorbate (with pretreatment DTT) and DHAsA (subtracting AsA from total ascorbate). This method was described by [67].

### 2.5. Statistics

Between four and five replicates were used for data analyses. Statistical analyses were carried out by two-way analysis of variances using SigmaPlot software. A two-way analysis of variance (ANOVA) was performed to test the independence of variation among conditions (equal variance test) and normal distribution of data of each variable (Shapiro–Wilk). The Holm–Sidak method (all pairwise multiple comparison procedures) was used to check whether the means of the posterior homogeneous subgroups differed significantly at *p* < 0.05.

## 3. Results

### 3.1. Growth

**Figure 1:**
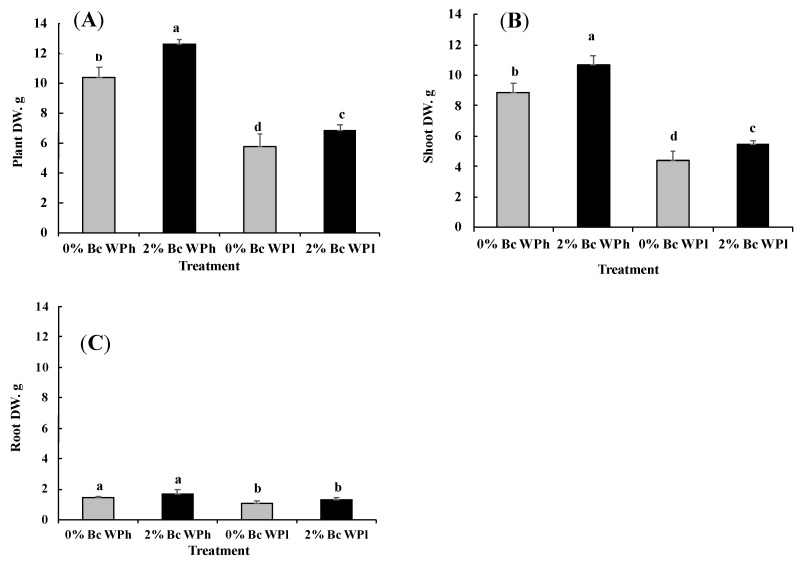
Varietal differences in plant growth parameters; dry weight of the whole plant (**A**), shoot (**B**), and the root (**C**), in *Medicago ciliaris* after 3 weeks of drought treatment. Values represent mean ± SE (*n* = 5) and the different letters a to d indicate significant differences between the treatments. Low soil water potential (WPl), high soil water potential (WPh), Biochar (Bc).

The dry weight of control plants (0% Bc WPh) was about 10 g at the time of harvest. Biochar amendment significantly increased the biomass (~24%), (Figure 1A). Instead, water deficit led to a significant reduction. However, this reduction was more pronounced in 0% Bc (~45%) than in 2% Bc.

Shoot and root dry weight variations were in the mean similar to those of the whole plant dry weight (Figure 1B,C).

The negative values of the sensitivity index (SI) under water deficit conditions reflect a growth-reduction (Table 1) and the positive values of SI at 2% Bc reflect a growth stimulation even under water deficit conditions.

**Figure 2:**
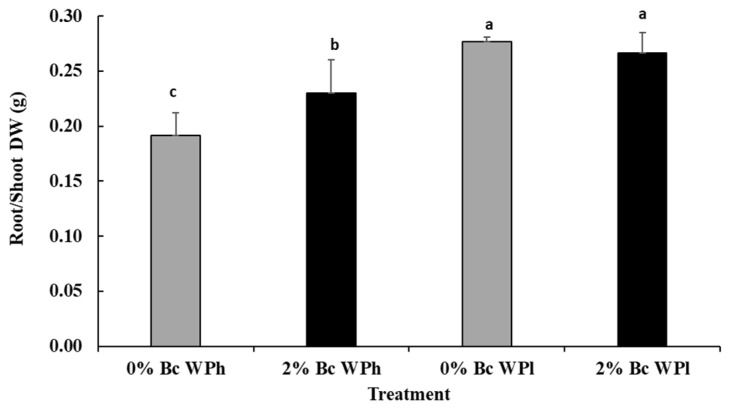
Root–shoot ratio in *Medicago ciliaris* after 3 weeks of drought treatment. Values represent mean ± SE (*n* = 5) and the different letters a to c indicate significant differences between the treatments. Low soil water potential (WPl), high soil water potential (WPh), Biochar (Bc).

As shown in Figure 2, the root/shoot ratio was significantly higher in plants subjected to water deficit stress than in controls. The biochar amendment caused a significant increase of the root/shoot ratio with sufficient water supply but not under drought conditions.

### 3.2. Tissue Water Status

**Figure 3:**
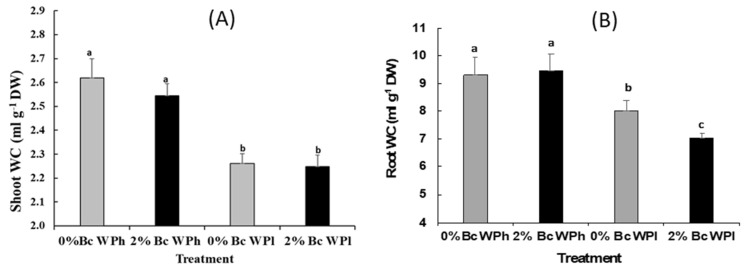
Shoot water content (**A**) and root water content (**B**) in *Medicago ciliaris* after 3 weeks of drought treatment. Values represent mean ± SE (*n* = 5) and the different letters a to c indicate significant differences between the treatments. Low soil water potential (WPl), high soil water potential (WPh), Biochar (Bc).

As shown in (Figure 3A), shoot and root water content were significantly reduced by water deficit by 55% and 27%, respectively, indicating the depressive effects of drought on water status in *Medicago ciliaris*. Biochar had hardly any effect on the water status with the exception of a further reduction in water content in the roots (Figure 3B).

### 3.3. Chlorophyll and Protein Content

**Figure 4:**
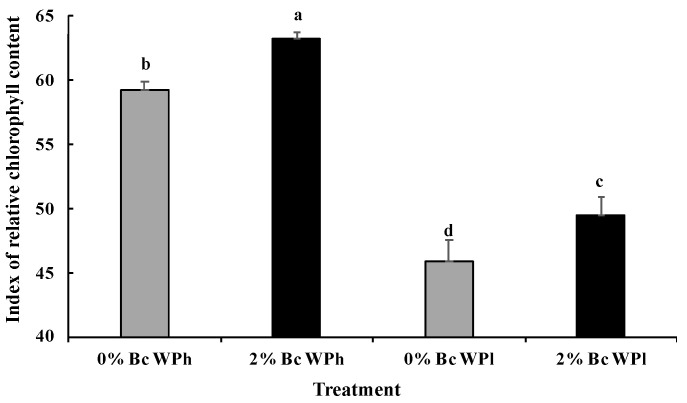
Chlorophyll concentration in *Medicago ciliaris* after 3 weeks of drought treatment. Values represent mean ± SE (*n* = 5) and the different letters a to d indicate significant differences between the treatments. Low soil water potential (WPl), high soil water potential (WPh), Biochar (Bc).

Water deficit stress significantly reduced chlorophyll content (Figure 4). The biochar amendment caused a significant increase in chlorophyll content at low and high soil water potential.

**Figure 5:**
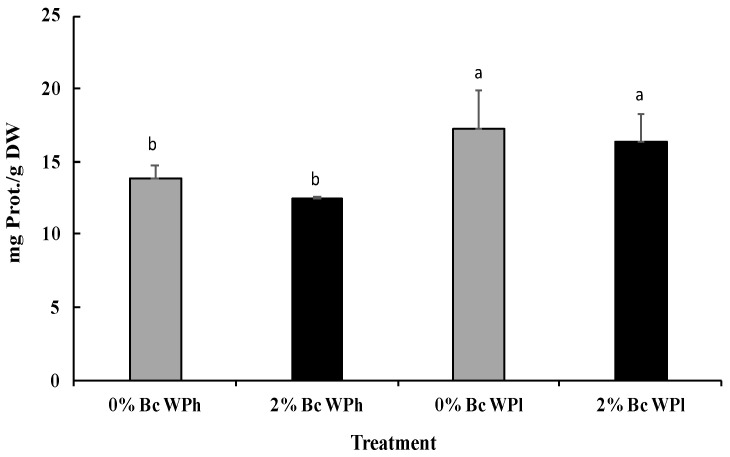
Leave protein content in *Medicago ciliaris* after 3 weeks of drought treatment. Values represent mean ± SE (*n* = 5) and the different letters a and b indicate significant differences between the treatments. Low soil water potential (WPl), high soil water potential (WPh), Biochar (Bc).

The development of the protein content was nearly reciprocal to the chlorophyll content (Figure 4 and Figure 5). Drought led to a significant increase of the protein content and biochar to a non-significant decrease (in the mean).

### 3.4. Proline and MDA Accumulation

**Figure 6:**
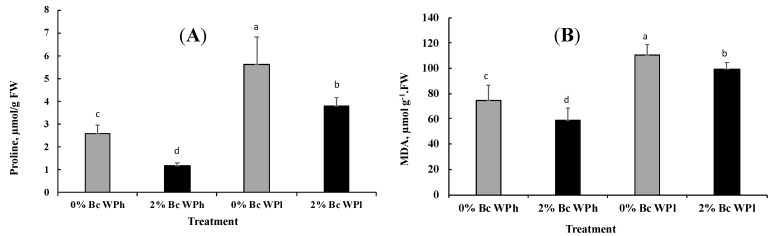
Changes in Proline (**A**) and MDA (**B**) content in *Medicago ciliaris* leaves after 3 weeks of drought treatment. Values represent mean ± SE (*n* = 5) and the different letters a to d indicate significant differences between the treatments. Low soil water potential (WPl), high soil water potential (WPh), Biochar (Bc).

Drought led to a significant increase of the proline content (Figure 6A) and of the MDA content (Figure 6B). However, biochar caused a significant decrease of the proline and MDA content at low and high soil water potential.

### 3.5. Leaf CO_2_/H_2_O Gas Exchange

The highest A_net_ was reached at a high water potential without biochar amendment (10.66 µmol m^−2^ s^−1^) (Table 2). Drought led to a significant decrease in A_net_ (2513 µmol m^−2^ s^−1^). The Biochar amendment significantly buffered the drought-induced reduction of A_net_. The differences in A_net_ correlated well with the photosynthetic efficiency (V_c_).

There was a clear correlation between the drought-induced reduction of A_net_ and ETR under the incorporation of dark and light respiration rates (the latter not shown) and a homeostatic and stable ETR/A_gross_ ratio in all four treatments.

There was a clear direct correlation between the drought-induced reduction of A_net_ and the stomatal conductance, with the logical consequence of low C_i_/C_a_ ratios in both generously watered treatments and high C_i_/C_a_ ratios in both water deficient treatments.

The application of biochar did not have any effect on C_i_/C_a_ and Sc. However, biochar had a significant positive impact on the water use efficiency at low water potential. This effect was reached mainly by the maintenance of high A_net_ rates.

### 3.6. Enzymatic Antioxidant Assays

**Figure 7:**
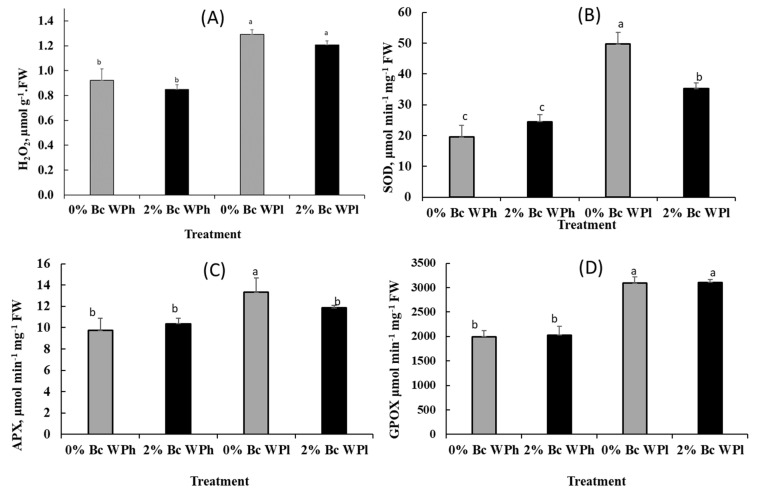

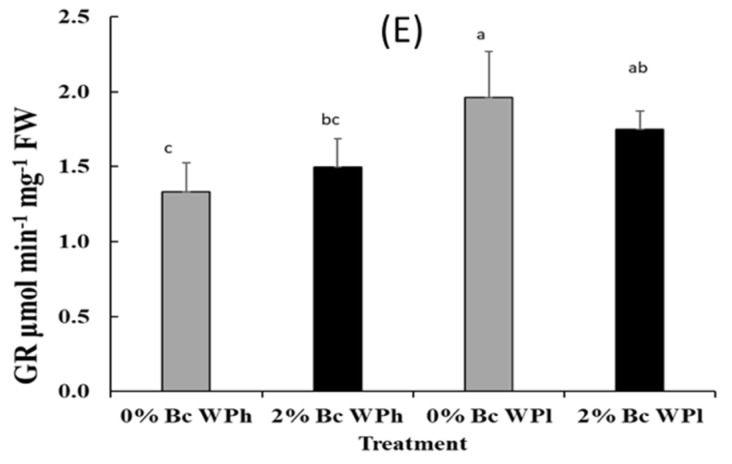
Varietal differences in the content of H_2_O_2_ (**A**) and the enzymatic activities of SOD (**B**), APX (**C**), GPOX (**D**), and GR (**E**) in *Medicago ciliaris* leaves after 3 weeks of drought treatment. Values represent mean ± SE (*n* = 5) and the different letters a to c indicate significant differences between the treatments. Low soil water potential (WPl), high soil water potential (WPh), Biochar (Bc).

Drought stress caused an increased accumulation of H_2_O_2_ in the leaves of *Medicago ciliaris* treated with and without biochar (Figure 7A). It is noticeable that higher H_2_O_2_ values correlate with lower chlorophyll content (see Figure 4) and lower photosynthetic activity (see Table 2). The drought induced an increase in reactive oxygen species (ROS), such as H_2_O_2_, making it necessary to also measure the antioxidant enzyme activities.

The drought-induced adjustment of *Medicago ciliaris* to an enhanced attack by reactive oxygen species (ROS) is reflected by increased activities of SOD, APX, GPOX and GR (Figure 7B–E). However, biochar reduced the APX and SOD activities in plants. Both enzymes constitute first line of defense against oxidative stress. Their reduced activities might be an indicator of a reduced demand for an adaptive response to ROS. The non-significant reduction of H_2_O_2_ content and GR activity points in the same direction.

### 3.7. Non Enzymatic Antioxidant Assays: Ascorbate Determination

**Figure 8:**
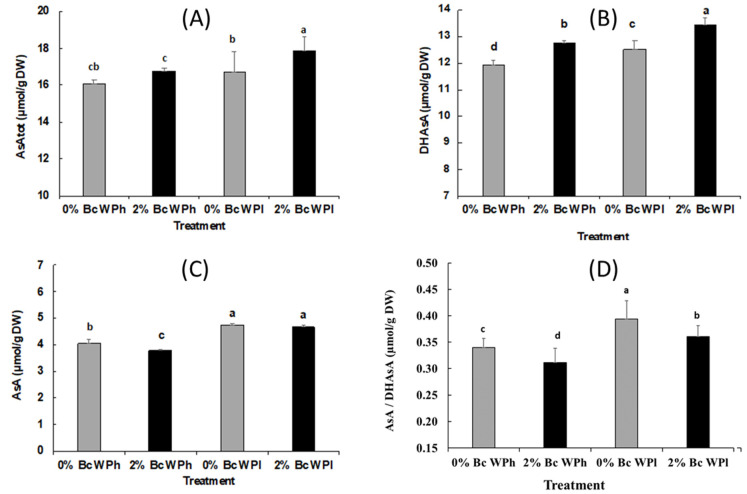
The content of total ascorbate (AsA + DHAsA) (**A**), AsA, (**B**), DHAsA (**C**), and AsA/DHAsA ratio (**D**) in *Medicago ciliaris* leaves after 3 weeks of drought treatment. Values represent mean ± SE (*n* = 5) and the different letters a to d indicate significant differences between the treatments. Low soil water potential (WPl), high soil water potential (WPh), Biochar (Bc).

Both factors water deficiency and biochar induced a significant increase in the total ascorbate concentration in the leaves of *Medicago ciliaris* (Figure 8A). Drought reached this effect by a joint increase of the reduced ascorbate (AsA, Figure 8B) and oxidized ascorbate (DHAsA, Figure 8C) concentration. However, biochar had a significantly higher impact on the concentration of DHAsA in drought, leading to an overall significant decrease in the AsA/DHAsA ratio in both biochar treatments (Figure 8D)

## 4. Discussion

In agreement with the present study, scientists worldwide are exploring possibilities in order to create the best possible growing conditions for drought-resistant crops that are able to maintain high productivity even in dry wastelands [68,69]. We decided to select *Medicago ciliaris* because it is a relatively salt-resistant alfalfa species.

### 4.1. Adjustment of Growth and Water Relations

However, it was obvious that alfalfa reduced biomass production by 45% in cases of water deficit (Figure 1). This result matches with results from [70], showing that the reduction of plant biomass production of medic plants ranged between 12 and 73%. However, it could be shown that the addition of Bc (biochar) to the soil substrate led to a significant increase in the biomass production of M. *ciliaris* in both water regimes (see also Sensitivity index in Table 1). Similar Bc effects were reported for rice and maize grain yields (increase of 12.1% and 28% respectively) [71,72,73].

Independent of the presence of Bc in the culture medium and in line with our findings, drought-stressed plants exhibit a higher root/shoot DW ratio than plants growing under adequate water supply (Figure 2). This may be related to the preferential allocation of dry matter to roots [70] and may facilitate adaptation to drought by limiting the transpiring leaf area and extracting water residuals [74].

The drought-induced increase of the root/shoot ratio may be also a consequence of decreasing RWC in both organs (Figure 3). The decrease of the RWC in case of water shortage seems to be a typical response of alfalfa. For instance, the exposure of several annual *Medicago spp*. *(M. rugosa, M. scutellata, M. littoralis, M. truncatula, M. murex, M. polymorpha, M. intertexta, M sativa*) to five days of drought led to a reduction of RWC up to 40% [75]. In the cases of *Medicago ciliaris* varieties, the decrease amounted to around 60% in severely dehydrated plants [76].

From this perspective, it is surprising that Bc amendment led not only to a further reduction of the root RWC during water shortage but also to higher growth rate. This apparent contradiction can be explained by the enhanced osmotic adjustment with organic osmoprotectants as a strategy to tolerate the adverse effects of drought conditions [74]. Furthermore, Bc improved soil quality by producing higher organic matter that enhanced growth-regulating substances and plant functioning [77].

Similar results were also found for soybean [49]. The authors reported that Bc application significantly enhanced crop growth rate, increased total biomass production approximately twice and improved WUE but did not improve RWC, water retention or uptake capacity in leaves. The assumption seems obvious that the enhanced biomass production after addition of Bc to the soil substrate may be caused by an improvement in plant nutrition rather than by increasing water uptake. Indeed, biochar application increased soil-available potassium (K) and enhanced its uptake and the stress resistance in soybean.

### 4.2. Regulation of Photosynthesis

The establishment of a new, suitable equilibrium and a high efficient use of resources is the main strategy during adaptation to water shortage in *M. ciliaris*. The following adaptation of photosynthesis to a new optimum is a good example of coordinated regulation in *M. ciliaris*: The suppressive impact of drought on photosynthesis (A_net_, Table 2) contributed, together with a reduction of the chlorophyll content (Figure 4), leaf area and photosynthetic electron transport rate (ETR), to the maintenance of a constant ETR/A_net_ ratio (Table 2). This mechanism reduced the generation of ROS and counteracted the otherwise possible destruction of chloroplasts [78,79].

Usually, a reduced chlorophyll concentration would imply a reduced ability for light harvesting and thus reduced photosynthesis [80]. *M. ciliaris* seems to actively use this correlation in case of drought or be forced into backwards regulation and the protection of the plants against oxidative stress, as confirmed in the current study. The latter process could happen because of the limited stomatal conductance and CO_2_/H_2_O gas-exchange during water shortage [81]. Indeed, our studies showed that A_net_ and S_C_ but not C_i_ (or the C_i_/C_a_ ratio, Table 2) increased and decreased simultaneously. Stomatal conductance was higher in plants receiving normal irrigation than in drought-treated plants [78]. The increase of the C_i_/C_a_ ratio in the leaf intercellular is a very common response during times of limited water supply because of reduction of flow through the closing stomates [82]. However, the opposite happened in *M. ciliaris* (Table 2). This can be explained by the fact that the photosynthetic carbon assimilation capacity decreased under water shortage to a higher degree (76%) than the leaf conductance (70%), which nicely illustrates the resultant photosynthetic shifts from stomatal to non-stomatal limitations. These results are similar to some earlier findings published by He et al. [83], where drought stress was also accompanied by increasing intercellular CO_2_ concentrations of bamboo leaves (*D. minor var. amoenus).* In this context it was shown that photosynthesis is primarily affected during mild and moderate stress conditions by stomatal limitation, but under severe water deficiency, it is affected by non-stomatal limitation in chloroplast CO_2_ fixation abilities, rather than CO_2_ diffusion resistance [84,85,86,87].

It is well known that Bc improves plant performance in the form of higher organic matter production, an increased synthesis in growth-regulating substances and an improved plant functioning [77]. Bc amendment also supports at water shortage, increasing the activity of anti-oxidant enzymes and the maintenance of high leaf chlorophyll content [88,89]. A number of previously published reports [46,90,91] showed that Bc application can improve soil water availability in general and buffer the effect of reduced water supply on plant photosynthetic carbon assimilation capacity. This is in line with our findings that soil amendments with Bc boost chlorophyll content both under well-watered and insufficiently watered plants. In comparison to the results of the water shortage treatment (0% Bc WP_l_, see above) and in agreement with the above-cited literature was the Bc-induced higher maintenance (2% Bc) of net photosynthesis (A_net_ only 40% less as WP_h_, Table 2) accompanied by a proportionally higher chlorophyll content (Figure 4, [78,92,93]) and a less pronounced decrease in stomatal conductance (S_c_, only 44% less as WP_h_). In order to survive in dry arid zones, optimizing photosynthesis as well as stomatal conductance is essential for plant species with the aim of preserving net CO_2_ assimilation and reduce evaporation [94]. Under these unfavorable conditions it seems to be beneficial that Bc application enhanced WUE in cases of water shortage (Table 2). The high WUE correlated with the maintenance of a high A_net_ and the reduction of non-stomatal limitation. By increasing WUE, oxidative stress is most likely reduced and, consequently, resistance against drought stress is increased (H_2_O loss per net CO_2_ uptake) [46]. This interpretation is in line with some previous studies in which soil amendment with Bc alleviated drought stress symptoms by significantly enhancing the water use efficiency, stomatal conductance, chlorophyll contents and photosynthesis of tomato, cowpea and okra leaves during water shortage [95,96].

A remarkable feature of the photosynthetic apparatus is its ability to adapt to changes in environmental conditions by sensing light quality and quantity, CO_2_ levels, temperature and nutrient availability [97]. The water shortage and the resulting low A_net_ and low WUE led in *Medicago ciliaris* (0% Bc) to a reduced demand and the necessity of the regulation of light energy coming through the chloroplast electron transport chain. While light is essential for photosynthesis, it can also lead to light-induced damage when the absorbed light energy exceeds the capacity of the photosynthetic machinery. To avoid that, the excess photons and electrons need to be dissipated. This occurs through photoinhibition or a rapidly inducible non-photochemical quenching process Y(NPQ) in which the absorbed excess light energy is dissipated as heat [98,99]. Chlorophyll fluorescence is an important photosynthetic parameter that reflects the absorption and utilization of light energy from Photosystem II (PSII). However, *M. ciliaris* does not respond to water shortage as expected with higher Y(NPQ) (Table 2) but rather with a significant decrease in ETR (*p* < 0.05) together with an increase in dark-respiration (R_D_) and decrease in light-respiration (R_L_), leading finally to no significant change in the ETR/A_gross_ ratio. The reduction in ETR correlates in *Medicago ciliaris* with a reduction of the chlorophyll content, indicating that PSII had been damaged to varying degrees, photosynthetic organs had been altered, and the effects of excessive light energy could not be disposed through heat dissipation (Y(NPQ)) but instead through photochemistry by producing large amounts of reactive molecules, causing oxidative damage to photosynthetic organs [100] and the resultant photosynthetic shifts from stomatal to non-stomatal limitation. In the current study, we applied Bc treatment in order to increase productivity and escape ROS damage. Indeed, biochar application during water shortage led to significant higher chlorophyll concentrations, higher A_net_ and also lower R_D_ and R_L_ (Table 2), giving the overall picture of a moderate stress response.

### 4.3. Indicators of Oxidative Stress

The results of CO_2_/H_2_O gas exchange and PSII chemistry confirms the acceptance of a possible increasing of oxidative stress during water deficiency in soil without Bc amendment, resulting in malfunctioning and eventually the death of the affected cells [101]. In general, several photoprotective mechanisms exist, such as plastid antioxidant enzymes and molecules [98] and repair processes for damaged PSII [102] and lipid peroxidation [97]. In this study we used MDA, proline and H_2_O_2_ as biomarkers for oxidative stress (MDA, Figure 6) [103], non-enzymatic photo-protection (proline, Figure 6) [104] and redox regulation (H_2_O_2_, Figure 7) [105].

In agreement with our expectations, water shortage led to a significant increase in MDA content in *M. ciliaris*, indicating a substantial stress by reactive oxygen species, leading to lipid peroxidation, fatty acid saturation and consequently damage to the membranes [106,107]. The formation of MDA is actually the consequence of enzymatic breakdown in cells. *M. ciliaris* plants grown with Bc amendment had lower MDA content than non-treated ones. This effect can be explained by the coordinated activation of protective enzymes (see Figure 7) which leads to attenuate ROS production, hence oxidative stress [90,108]. Our results are similar to some earlier findings in which soil amendment with Bc decreased the MDA content of *Phragmites karka* and *Brassica olerecae* (Cabbage seedling) under drought stress conditions [109,110].

We got similar responses of both proline and MDA as to Bc amendment during water stress. Proline, produced under stressful conditions, can act as a free radical scavenger for photo-protection but also a compatible solute in osmotic adjustment [111]; a metal chelator; an activator of ROS detoxification pathways; a cell redox balancer; a cytosolic pH buffer; a source of energy; a source of nitrogen and carbon; a stabilizer of subcellular structures and membranes, including photosystem II [112]; and can act as a signaling molecule [104]. Water shortage in *M. ciliaris* leaves (0% Bc) led to a considerable increase in leaf proline accumulation, which shows the importance of proline as photoprotectant and osmoprotectant (see also [113,114]. In agreement with our findings, Yildirim [110] reported that Bc treatment lowered proline content in the plants. Our results suggest reduced osmotic and oxidative stress in Bc-treated plants.

The last used biomarker, H_2_O_2_, regulates plant growth, development and acclimatory and defense responses [115]. Moreover, among oxidative species, H_2_O_2_ is a very vigorous metabolite that deteriorate the structure of biological membranes during abiotic stresses [116]. The increased production of ROS (such as H_2_O_2_) in drought-stressed plants of *M. ciliaris* (Figure 7) is a common phenomenon taking place under stress conditions [117]. Plants use antioxidant defense (enzymatic or non-enzymatic) to deal with oxidative stress [90]. Plants employ diverse defensive adaptive mechanisms to survive under adverse cues such as the activation of a signaling pathway, expression of genes and accumulation of stress-related proteins [118] and enzymes. The latter effect may also be used to explain the increase in protein content in *Medicago ciliaris* plants subjected to water shortage (Figure 5).

### 4.4. Photoprotective Mechanisms: Enzymatic Oxidants

In the present investigation, water shortage boosted enzymatic and non-enzymatic antioxidant activity in *M. ciliaris* plants. Enzymes like superoxide dismutase (SOD), ascorbate peroxides (APX), guaiacol peroxidase (GPOX) and glutathione reductase (GR) are directly engaged in catalyzing ROS degradation reactions by directly scavenging ROS [103,119] and indirectly reducing membrane lipid peroxidation and alleviating the damage in PSII structure and function.

The first enzyme in the antioxidant pathway is SOD, which removes superoxide radical by catalyzing its dismutation to H_2_O_2_ and another oxidized to O_2_ [120]. The increase in SOD activity observed in the leaves of *M. ciliaris* (Figure 7) as a function of the applied water stress levels might be correlated to the enhanced protection from damages, among them lipid peroxidation, associated with oxidative stress. In *M. ciliaris*, water shortage led to increased concentrations of APX, GPOX and GR (see Figure 7), suggesting the involvement of the Halliwell–Asada pathway, where APX reduces H_2_O_2_ to water and MDHA using ascorbic acid as substrate [121] at the expense of NADPH [26,122,123]. Khaleghi [103] reported that APX activity increased in drought-stressed *Maclura pomifera*, *Picea aspertata* and *Nicotiana tabacum*, respectively. The stimulation of APX activity might be correlated to the increased H_2_O_2_ generation by the observed enhanced SOD activity (both Figure 7). Wang [124] reported that peroxidase activity is closely related to PSII electron transport properties and PSI, but the activity of the latter could be inhibited with the increase in SOD activity.

SOD and APX showed maximum activity in the leaves of untreated plants and minimum activity in the leaves of Bc-treated plants (Figure 7). These results correlate with the Bc-mediated decrease in MDA and proline contents in the leaves and an improvement of photosynthetic parameters. It was previously shown that Bc application can buffer the impact of drought on photosynthetic apparatus by regulating the activity of protective enzymes and affecting electron transfer [86,90]. Our results are similar to some earlier findings in water shortage conditions where Bc application lowered enzymatic activity and lipid peroxidation and enhanced photosynthesis in *Pyrus ussuriensis* Maxim [90] *Brassica olerecae* [110] and *Phragmites karka* [109].

### 4.5. Photoprotective Mechanisms: Non-Enzymatic Antioxidants

Besides enzymatic antioxidants, there are non-enzymatic antioxidants, such as reduced (AsA) and oxidized (DHAsA) ascorbate, glutathione (GSH) and carotenoid, which can play a role in the antioxidant system in two ways, either directly interacting with ROS or functioning as substrates in enzyme-catalyzed ROS-degrading reactions [101,119,125]). Ascorbate (AsA, Vitamin C) is one of the universal non-enzymatic antioxidants, as it has the ability to donate a hydrogen atom and form a relatively stable ascorbyl-free radical. It protects plants against oxidative damage by environmental stresses, such as drought [34,126]. It participates in diverse redox and ROS neutralization reactions in the chloroplast and can be a facultative electron donor for the photosynthetic electron transport chain [127]. DHAR is responsible for regenerating AsA from the oxidized state and regulates the cellular AsA redox state, which is crucial in the response to abiotic stresses. Ascorbic acid (AsA) and its oxidized form dehydroascorbate (DHAsA) play a key role in redox state-based signaling mechanisms by the detoxification of ROS and its products, as well as the transmission of redox signals [128]. To prevent levels exceeding the anti-oxidative capacity of cells, ROS formation has to be closely regulated.

The increased activity of APX at water shortage correlates with the increase of AsA and DHAsA and can be explained by the high demand and capacity to eliminate H_2_O_2_ in the leaves of *M. ciliaris* (s.a.). An increased AsA–GSH cycle enables chloroplast to prevent photoinhibition by maintaining the NADP+/NADPH ratio so that ETR is least affected [129].

Water shortage causes a significant increase in DHAsA content (Figure 8). Several plant studies revealed that the upregulation of the AsA–GSH pathway enzymes and the enhancement of the DHAsA and GSSG levels gave plants better tolerance to abiotic stresses by reducing the ROS [130]. DHAsA is supposed to be involved in zeaxanthin biosynthesis by dissipating excess light energy in the thylakoid membranes of chloroplast and preventing oxidative stress by maintaining the activity of antioxidant enzymes. In accordance with our expectations, water shortage also led to a significant increase in AsA (reduced form of ascorbate) content and the AsA/DHAsA ratio. Alterations in the AsA/DHA ratio is involved in stress sensing, and redox homeostasis is one of the most important factors for protecting cells from ROS toxicity [131]. The leaf apoplast redox status specifically modulates plant growth and their response to hormones, antioxidant enzyme activities, expression patterns of catalase, glycolate oxidase and some other genes, and MAPK activity and the regulation of transcripts associated with calcium channels [132]. For instance, the AsA/DHAsA ratio is a prominent modulator of the enzymes responsible for carbon assimilation under stress conditions [28] and usually calculated to evaluate AsA availability and, thus, used as an indicator of oxidative stress and initiators of the plant defense system [28]. Several authors mentioned that the increase in the AsA/DHAsA ratio above a distinct limit lead to an enhanced production of abscisic acid in plants [133] and could cause, in accordance with the available data shown in this paper, the closing of stomata, the reduction of CO_2_ fixation, cell expansion and plant growth (see [134]). Moreover, the increase of the AsA/DHAsA ratio at 0% Bc and water shortage up to the highest level of all four treatments deepens the impression that the increase in SOD, APX, GPOX and GR activities were not high enough to hinder extensive ROS damage in *M. ciliaris* leaves.

However, soil amendment with biochar (2% Bc) during water shortage led to a significant reduction of the AsA/DHAsA ratio down to the level of well-watered control plants (0% Bc). This Bc-mediated effect was mainly reached by the significant higher increase in both the total ascorbate AsA_tot_ (oxidized and reduced) and DHAsA content, which optimize *M. ciliaris* chances in buffering oxidative stress by directly scavenging ROS [66,131].

## 5. Summary and Conclusions

The results presented in this study support our assumption that Bc application enhances the performance of *M. ciliaris* during times of limited water supply. Indeed, biochar application during water shortage led to a more moderate exposure to water deficiency and in this way supported an improved and interactive plant adjustment. It led to a reduced impact of limited water supply on growth and water relations and included a balanced regulation of photosynthesis and the buffering of reactive oxygen species with photo-protective mechanisms. In latter case, the results impressively confirmed the agreement with the enzymatic ROS defense by the Halliwell–Asada pathway enzymes (Figure 7), corresponding to our previous statement that Bc application significantly reduces the negative impact of reactive oxygen species on *M. ciliaris* plants. This aspect is evidenced by the fact that the Bc-mediated moderate change of the AsA/DHAsA ratio also lead to only the minor stimulation of abscisic acid production [133]. This interpretation is also confirmed indirectly by the response of the CO_2_/H_2_O gas exchange parameters (Table 2).

We conclude that amendment through Bc may be a helpful approach to improve the performance of *Medicago ciliaris* during water shortage and to increase plant productivity in the arid land regions. This species seems to be suited to use a cash pasture plant in the development of agriculture on dry wasteland in a future world of water shortage. However, further field trials should be carried out under corresponding conditions as an intermediate step before agricultural use to verify the presented results on an agro-ecosystem level.

## Figures and Tables

**Table 1:** Relative impact (SI in %) of drought and biochar on the dry weight of *Medicago ciliaris* plant, shoot and root. Low soil water potential (WPl), high soil water potential (WPh), Biochar (Bc).

Sensitivity Index (SI)	WPl	Bc WPh	Bc WPl
Plant	−46.81%	17.61%	−37.2%
Shoot	−50.47%	40.29%	−37.77%
Root	−24.34%	16.38%	−9.00%

**Table 2:** CO_2_/H_2_O gas exchange and chlorophyll fluorescence parameters (A_net_, V_c_, SC, C_i_/C_a_ ratio, WUE, R_L_, R_D_, ETR, ETR/A_gross_ and Y(NPQ)) of *Medicago ciliaris* leaves at a saturating light intensity after 3 weeks of drought treatment. Values represent mean ± SE (*n* = 5) and different letters indicate significant differences between treatments. Net CO_2_ assimilation rate (A_net_) [μmol(CO_2_) m^–2^ s^–1^], photosynthetic efficiency (V_c_), stomatal conductance (S_c_) [mol(H_2_O) m^–2^ s^–1^], ratio of intercellular and atmospheric CO_2_ concentration (Ci/Ca ratio) [μmol(CO_2_) m^–2^ s^–1^], ratio of net CO_2_ assimilation rate and transpiration (A/E) (µmol/mmol), Photorespiration (R_L_) (µmol(CO_2_)*m^−2^*s^−1^), dark respiration (R_D_) (µmol CO_2_ m^−2^ s^−1^), electron transport rate (ETR) (µmol electrons m^−2^ s^−1^), gross CO_2_ assimilation (A_gross_) [μmol(CO_2_) m^–2^ s^–1^], quantum yield of regulated non-photochemical energy loss in PS II (Y(NPQ)), electron (e^−^).

Treatment Parameter	WPh (at 1500 μE m^−2^ s^−1^ PPFD)		WPl (at 750 μE m^−2^ s^−1^ PPFD)	
	0% Bc	2% Bc	0% Bc	2% Bc
A_net_ (µmol CO_2_ m^−2^ s^−1^)	10.667 a ± 0.566	8.029 b ± 0.803	2.513 d ± 0.294	4.495 c ± 0.801
V_c_ (µmol CO_2_*m^−2^*s^−1^)	0.057 a ± 0.008	0.048 ac ± 0.012	0.030 b ± 0.001	0.045 bc ± 0.012
S_C_ (µmol CO_2_ m^−2^ s^−1^)	0.07 a ± 0.008	0.05 b ± 0.004	0,021 c ± 0,008	0.03 c ± 0.007
C_i_/C_a_ ratio	0.367 a ± 0.05	0.323 a ± 0.06	0.510 b ± 0.09	0.422 b ± 0.01
WUE (A/E)	9.476 b ± 1 27	6.595 c ± 0.21	6.4 d ± 1.85	11.04 a ± 0.61
R_L_ (µmol(CO_2_)*m^−2^*s^−1^)	11.88 b ± 0.73	13.59 a ± 0.95	9.05 c ± 0.56	8.01 c ± 0.72
R_D_ (µmol CO_2_ m^−2^ s^−1^)	1.189 ab ± 0.141	0.640 b ± 0.157	1.675 a ± 0.600	0.900 b ± 0.452
ETR (µmol e^−^ m^−2^ s^−1^)	182.23 b ± 7.71	195.73 a ±8.15	112.38 c ± 3.9	109.6 c ± 6.42
ETR/A_gross_ ratio (e^−^/CO_2_)	7.75 a ± 0.2	8.65 a ± 0.26	8.43 a ± 0.63	8.4 a ± 0.68
Y(NPQ)	0.382 a ± 0.04	0.301 b ± 0.06	0.329 a ± 0.03	0.371 a ± 0.029

## Data Availability

Not applicable.

## Abbreviations

Biochar (Bc), fresh weight (FW), dry weight (DW), water-holding capacity (WHC), net CO_2_ assimilation rate (Anet), photosynthetic efficiency (Vc), stomatal conductance (Sc), ratio of intercellular and atmospheric CO_2_ concentration (Ci/Ca ratio), ratio of net CO_2_ assimilation rate and transpiration (A/E), photorespiration (RL), dark respiration (RD), electron transport rate (ETR), gross CO_2_ assimilation (Agross), quantum yield of regulated non-photochemical energy loss in PS II (Y(NPQ)), electron (e^−^), photosystem 2 (PSII), non-photochemical quenching (NPQ), cycling electron flow (CRF), reactive oxygen species (ROS), ascorbate (reduced form) (AsA), dehydroascorbate (oxidized form) (DHAsA), glutathion (GSH), sensitivity index (SI), hydrogen peroxide (H_2_O_2_), malondialdehyde (MDA), superoxide dismutase (SOD), ascorbate peroxidase (APX), guaiacol peroxidase (GPOX), glutathione reductase (GR).
